# Randomized trial of two artificial intelligence coaching interventions to increase physical activity in cancer survivors

**DOI:** 10.1038/s41746-021-00539-9

**Published:** 2021-12-09

**Authors:** Ahmed Hassoon, Yasmin Baig, Daniel Q. Naiman, David D. Celentano, Dina Lansey, Vered Stearns, Josef Coresh, Jennifer Schrack, Seth S. Martin, Hsin-Chieh Yeh, Hadas Zeilberger, Lawrence J. Appel

**Affiliations:** 1 Johns Hopkins Bloomberg School of Public Health, 615 N. Wolfe Street, Baltimore, MD 21205 USA; 2 Johns Hopkins University School of Medicine, 733 N. Broadway, Baltimore, MD 21205 USA; 3 Welch Center for Prevention, Epidemiology, & Clinical Research, Johns Hopkins University, 2024 E. Monument Street, Baltimore, MD 21205 USA; 4 University of Michigan Medical School, Ann Arbor, MI USA; 5 Department of Applied Mathematics and Statistics, Whiting School of Engineering, Johns Hopkins University, 3400 North Charles Street, Baltimore, MD 21218-2608 USA

**Keywords:** Lifestyle modification, Randomized controlled trials, Cancer prevention

## Abstract

Physical activity (PA) has numerous health benefits. Personalized coaching may increase adherence to PA recommendations, but it is challenging to deliver personalized coaching in a scalable manner. The objective of our study was to determine whether novel artificially intelligent (AI) coaching interventions increase PA among overweight or obese, physically inactive cancer survivors compared to a control arm that receives health information. We conducted a single-center, three-arm randomized trial with equal allocation to (1) voice-assisted AI coaching delivered by smart speaker (MyCoach), (2) autonomous AI coaching delivered by text message (SmartText), and (3) control. Data collection was automated via sensors and voice technology, effectively masking outcome ascertainment. The primary outcome was change in mean steps per day from baseline to the end of follow-up at 4 weeks. Of the 42 randomized participants, 91% were female, and 36% were Black; mean age was 62.1 years, and mean BMI was 32.9 kg/m^2^. The majority were breast cancer survivors (85.7%). At the end of 4 weeks follow-up, steps increased in the MyCoach arm by an average of 3618.2 steps/day; the net gain in this arm was significantly greater [net difference = 3568.9 steps/day (95% CI: 1483–5655), *P* value <0.001] compared to control arm, and [net difference = 2160.6 steps/day (95% CI: 11–4310), *P* value 0.049] compared to SmartText. In conclusion, AI-based voice-assisted coaching shows promise as a practical method of delivering scalable, individualized coaching to increase physical activity in sedentary cancer survivors. Additional research is needed to replicate these findings in a broader population of cancer survivors and to investigate the effects of these interventions in the general population.

**ClinicalTrials.gov Identifier:** NCT03212079, July 11, 2017, https://clinicaltrials.gov/ct2/show/NCT03212079.

## Introduction

Despite obesity’s well-documented association with poor health outcomes—including increased risk of cardiovascular disease (CVD), diabetes, and cancer incidence and recurrence—the prevalence of overweight and obesity has risen sharply over the past several decades^1^. Nearly 42.4% (2017–2018 NHANES) of US adults have obesity^1^, with an estimated annual economic burden of US$190 billion in total costs, corresponding to 21% per year of total direct healthcare spending in the US^2^. In recent years, CVDs became one of the leading causes of death among cancer survivors^3^. Obesity among cancer survivors significantly reduces survivorship^4^. Increasing physical activities among cancer survivors significantly reduced all-cause mortality^5^. Behavioral approaches to reduce calorie intake and increase physical activity are essential features of weight loss interventions, which typically involve individualized, person-to-person coaching^6,7^. However, such programs are time- and cost-intensive^8^.

The introduction of intelligent voice assistance using smart devices—phones, wearables, in-home speakers, the ability to deploy learning models and other technologies comprising the Internet of Things—is a potential way to deliver behavioral base interventions closer to the classic model of person-to-person coaching but at lower cost and in a scalable form. The potential scalability of AI interventions is especially important given the extremely high prevalence of physical inactivity and limited reimbursement for lifestyle counseling. For these reasons, the classical model of “person-to-person,” behavioral interventions are insufficient as a public health response given the scope of the problem. The ability of artificially intelligent agent to generate adaptive responses based on behavior, language process for human-machine interaction, and on-demand data analytics in the cloud present an opportunity to create an artificially intelligent agent that can provide instant, individualized, on demand health coaching adapted by user’s behavior. Such coaching has the potential to calibrate guidance based on real-time data from a user’s fitness tracker and health records.

Our team designed and developed two artificial intelligent (AI)-based, physical activity interventions and tested them in a three-arm, randomized, controlled pilot study. The AI-based health coaching agent (MyCoach) was delivered through an in-home smart speaker—a relatively recent technology found in nearly half of homes in the United States in 2018^9^. A second AI intervention was autonomous progressive smart coaching delivered through text messaging (SmartText). The third arm received educational materials on physical activity (control). The aim of our study was to determine whether novel AI coaching interventions increase physical activities among overweight or obese, physically inactive cancer survivors compared to a control arm that receives health information.

## Results

### Participants

Fourteen participants enrolled in each arm; participant flow from screening to enrollment is summarized in the consort figure (Supplementary Fig. 1). Table 1 provides participants’ characteristics by study arm and overall. Mean (SD) age was 62.1 (9.8) years and mean (SD) body mass index (BMI) was 32.9 kg/m^2^ (5.0). The majority of participants (85.7%) had stage 1 or 2 breast cancer. Baseline characteristics were similar in the study arms.

**Table 1:** Characteristics of the study participants by randomization arm.

Characteristics	All	Control	SmartText	MyCoach
Number of participants	42	14	14	14
Female, *n* (%)	38 (90)	14 (100)	11 (79)	13 (93)
Age in years, mean (SD)	62.1 (9.8)	63.9 (9.3)	64.1 (7.2)	58.1 (11.8)
Body mass index (kg/m^2^), mean (SD)	32.9 (5.0)	35.2 (5.8)	31.4 (3.7)	32.1 (4.1)
Overweight, *n* (%)	12 (28.6)	2 (14.3)	5 (35.7)	5 (35.7)
Obese, *n* (%)	30 (71.4)	12 (85.7)	9 (64.3)	9 (64.3)
Race (Black), *n* (%)	15 (36)	6 (43)	4 (29)	5 (36)
Cancer type, *n* (%)				
Breast	36 (85.7%)	13 (93)	11 (79)	12 (86)
Prostate	1 (2.3%)	—	1 (7)	—
Lung	2 (4.7%)	1 (7)	1 (7)	—
Colon	1 (2.3)	—	1 (7)	—
Other	2 (4.7)	—	—	2 (14)
Stage of cancer, *n* (%)				
0	1 (2)	1 (7)	—	—
1	18 (43)	7 (50)	5 (36)	6 (43)
2	12 (29)	5 (36)	4 (29)	3 (21)
3	8 (19)	—	4 (29)	4 (29)
Unknown	3 (7)	1 (7)	1 (7)	1 (7)

### Physical activity

**Fig. 1:**
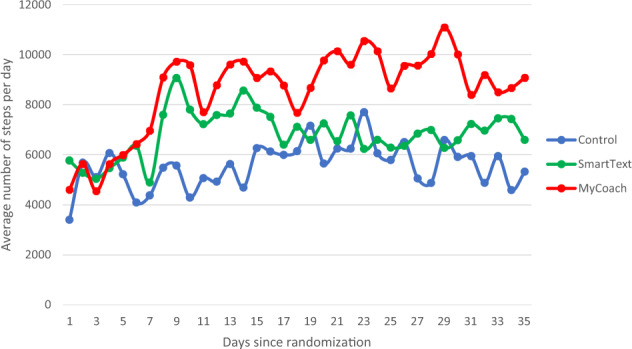
Average steps per day by groups. Blue line—control. Green line—SmartText. Red line—MyCoach.

At baseline, the average number of steps was similar in the three study arms (Table 2). Within each arm, the average number of steps increased between baseline and follow-up by a mean of 886.1 steps/day (95% confidence interval (CI): −895 to 2667) in the control arm, 1619.0 steps/day (95% CI: −328 to 3566) in SmartText arm, and 3618.2 steps/day (95% CI: 2490–4764) in the MyCoach arm (Table 2, top panel). When the fourth week of intervention was used alone as follow-up (Table 2, bottom panel), there were similar patterns, e.g., mean (95% CI) change in the MyCoach arm was 3585.0 steps/day (95% CI: 2304–4866). Figure 1 displays the average number of steps per day, by study arm, for each day post-randomization. In the MyCoach arm, there was an early and sustained increase in the average number steps, while in the SmartText arm, an early increase in steps per day was not sustained. During the whole intervention period, the number (%) of person/days achieved ≥10,000 steps per day was 28, 41, and 61% in the control, SmartText, and MyCoach arms, respectively. During the fourth week of follow-up only, corresponding results were 31, 34, and 58%.

**Table 2:** Change in average number of daily steps (follow-up minus baseline) within each arm.

Groups	Baseline mean (SD)	4-weeks of intervention mean (SD)	Within-group change mean (95% CI)
Change in the average number of steps from baseline to end of follow-up (all 4 weeks of intervention)			
Control	4847.0 (2925.7)	5733.1 (4399.9)	886.1 (−894.9, 2667.1)
SmartText	5522.4 (3528.3)	7141.4 (4459.1)	1619.0 (−328.1, 3566.2)
MyCoach	5683.8 (3194.2)	9302.0 (3307.1)	3618.2 (2490.1, 4764.2)
Change in the average number of steps from baseline to end of follow-up (last week of intervention)			
Control	4847.0 (2925.7)	5593.7 (4731.8)	746.6 (−1544.1, 3037.4)
SmartText	5522.4 (3528.3)	6924.8 (4181.3)	1402.4 (−1025.6, 3830.4)
MyCoach	5683.8 (3194.2)	9268.8 (2895.2)	3585.0 (2303.6, 4866.4)

The MyCoach arm achieved a greater increase in the average number of steps/days with an average net difference of 3568.9 steps/day (95% CI: 1483–5655, *P* value < 0.001) compared to control arm. MyCoach arm also achieved an average net difference of 2160.6 steps/day (95% CI: 11–4310, *P* value 0.049) compared to SmartText arm (Table 3, top panel). Results were similar after limiting the follow-up period to the fourth week of intervention (Table 3, bottom panel).

**Table 3:** Between-group differences: change from baseline to follow-up.

Groups	Change–change, mean (95% CI)	Change–change, *P* value
Between-group differences: change from baseline to follow-up (all 4 weeks of intervention)		
SmartText–control	1408.2 (−1312.4, 4128.9)	0.30
MyCoach–control	3568.9 (1482.7, 5655.0)	0.001
MyCoach–SmartText	2160.6 (11.4, 4309.7)	0.049
Between-group differences: change from baseline to follow-up (last week of intervention)		
SmartText–control	1331.0 (−1731.9, 4393.9)	0.39
MyCoach–control	3675.1 (1304.7, 6045.5)	0.003
MyCoach–SmartText	2344.1 (152.8, 4535.3)	0.037

### Intervention process data

The intervention was delivered as intended in all three study arms. All participants in the control arm received printed and emailed National Cancer Institute (NCI) educational materials about physical activities for cancer survivors. All SmartText participants received three text messages per day during the intervention period, except one participant who did not get messages on 1 day. MyCoach participants had an average of two interactions per day. Most of the voice interaction sessions concerned physical activity reminders and progress (66%), asking for health tips (23%), checking local resources (7%), and checking for sunscreen need before going out for a walk (4.3%).

### Safety

There were no adverse events during the intervention period.

## Discussion

In the Physical Activity by Technology Help (PATH) trial, we tested two novel AI-based interventions to increase physical activities among overweight or obese cancer survivors. Our main finding was that AI-based, voice-assisted coaching delivered through a smart speaker (MyCoach) significantly increased physical activity in comparison to the control arm and the AI-based autonomous texting (SmartText). In addition, while the SmartText intervention increased physical activity, the change in physical activity did not differ from the change in physical activity in the control arm.

While physical activity increased in each arm, the patterns over time appeared to differ. During the initial 2 weeks of the intervention period, participants in the control arm had a gradual rise in steps, followed by a gradual reduction over the last 2 weeks. Participants in the MyCoach and SmartText arms both experienced a spike in activity in the first week. However, those enrolled in the SmartText arm had a gradual reduction in activity during the following 3 weeks. In contrast, the MyCoach arm participants sustained a high level of physical activity throughout the intervention period. Potential reasons for the apparent differences in physical activity between SmartText and MyCoach interventions are manifold. One reason is the approach to communication between the AI agent and participant. The intervention in MyCoach is dependent on the participant’s desire to seek coaching that occurred in a bidirectional conversation, while the intervention in the SmartText arm provided coaching via text messages in a unidirectional fashion from the AI agent to the participant. Similarly, a prior study that aimed to increase physical activity among patients with diabetes showed sustained gain in physical activities for an extended period, 12 weeks, and used mobile app with bidirectional communication but powered by human coaching^10^. A second reason is the flexible vs fixed contact frequency in the MyCoach and SmartText arms, respectively. Nevertheless, both interventions used had similar content and goals. These results suggest that technology interventions that allow users to decide when and how to use the technology may have a better chance at achieving and sustaining increased physical activity and perhaps other lifestyle changes.

Our target population in this study was cancer survivors who were sedentary and overweight or obese. Increased physical activity coupled with weight reduction might benefit this group by reducing the risk of cancer recurrence and reducing morbidity and mortality from CVDs. In recent years, our research group has focused on weight loss in overweight and cancer survivors and has published the results of two trials^11–13^ and is conducting a third (NCT04534309). Still, the physical inactivity component of the interventions implements the classic “person-to-person” model, which is problematic given the need for scalability and concerns about effectiveness. Despite the high prevalence of overweight, obesity, and sedentary lifestyle, our results may not be generalizable to all sedentary people who are overweight or obese, as well as normal weight cancer survivors. The study interventions were specifically designed and tested for cancer survivors. However, similar approach can be adapted for other population in future studies. In addition, our study was a short-term pilot trial. Also, the trial was not designed to assess the impact of the interventions on weight change or general health. Our team is planning to assess these outcomes in a long-term study.

A systematic review of studies aimed to increase physical activities among adults with cancer using technology support concluded that most of the studies were of short duration without sufficient time to assess long-term outcomes^14^. Still, given abundance evidence on the benefit of increased physical activity on general health, and the concurrent public health burden of CVD, sedentary lifestyle, and overweight/obesity in cancer survivors as well as the general public, the need for practical, scalable interventions to increase physical activities is a public health priority.

In this context, AI technology has the potential to deliver effective interventions at scale to promote physical activity and perhaps other lifestyle interventions. Importantly, as AI-based interventions become widely available with large numbers of users, the learning and training of AI agents will likely improve. The use and effectiveness of AI agents (supervised or unsupervised) can be further enhanced through publicly accessible, open-access repositories with the learning algorithms. Some of the challenges to scale unsupervised AI intervention is computational resources. However, a recent study demonstrated an efficient use of computational resources to optimize model hyper-parameter tuning for fast physical activity recommendation in mobile health. Our study did not have to account for this challenge since the use of a dedicated server for the model computation is separate from the server that runs the voice interface^15^.

Our study has limitations. It was a pilot study with only 4 weeks of follow-up. However, in an analysis that included only the last week of follow-up, the MyCoach arm still showed a significant improvement in physical activity that highlights the potential for sustainability. Second, the trial enrolled a small number of participants. Replication of the trial with a large number of participants followed for a longer period of time is warranted. Third, the eligibility criteria of the trial included use of technology, which is not universally available now. However, digital voice assist technology is widely accepted and used by millions of users in the US^9^. Fourth, there are complex issues related to data security and privacy. At the time we developed these technologies, our institutional review board (IRB) required that we host and operate the AI agents from a secure server at Johns Hopkins University. However, technology companies now offer HIPAA compliant voice technology for such use, which makes AI technology interventions easily scalable.

Our study also has several strengths. Even though the trial was a pilot study, it was adequately powered and designed to detect changes in physical activity. Second, follow-up and data completeness were high; all participants had follow-up data, and the vast majority had completed primary outcome data. Third, trial conduct was extremely efficient with automated interventions, data collection, data transfer, and data storage. After the initial in-person visit, there were no other required in-person visits to the research clinic; all outcome data were collected remotely, mostly by physical activity sensor. In addition, recruitment was also efficient, using the electronic health record to target invitations, including mailings^16^.

In conclusion, AI voice-assisted coaching shows considerable promise as a practical method of delivering scalable, individualized coaching to increase physical activity in sedentary cancer survivors. Further research would be required to investigate whether this intervention produces similar results in the general population.

## Methods

### Design

The PATH study was a three-arm, randomized parallel trial with an allocation ratio of 1:1:1. A detailed description of the trial’s interventions has been published^16^. An IRB at Johns Hopkins University School of Medicine approved the trial protocol. All participants provided written informed consent.

### Eligibility

The principal eligibility criteria were: (1) Maryland adults with a history of breast, prostate, colon, lung, cervical, oral, or melanoma cancer; (2) overweight or obese as defined by BMI of ≥25 kg/m^2^; (3) completion of cancer treatment (surgery, chemotherapy, or radiation) at least 3 months before enrollment, with the exception of anti-hormonal therapy; (4) access to a smart phone (Android or OS); (5) ability to perform mild-to-moderate physical activity, such as walking; (6) physician clearance; (7) willing to be randomized to each study arm; and (8) willing to wear an activity tracker throughout the 5-week study period. The principal exclusion criteria were: (1) engagement in routine physical activity of ≥150 min per week during the 4 weeks before screening (using The Godin–Shephard Leisure-Time Physical Activity Questionnaire)^17^; (2) stage 4 cancer; (3) plans to re-locate during the study; (4) structured physical activity as part of a program, study, or consumer technology guide (e.g., Fitbit); (5) self-reported history of a psychiatric condition that may prevent the participant from performing study activities; and (6) pregnant or planning to become pregnant during the study period.

After screening and randomization, there was a 1-week baseline period followed by a 4-week intervention period. During the baseline period, we conducted safety monitoring and established baseline physical activity for each participant. Participants were instructed to not engage in any new activities outside of their current physical activities’ during the baseline period. The level of physical activities during baseline was not subjected to any exclusion criteria. After the baseline period, the interventions began.

### Measurements

At an initial visit, we obtained written informed consent, assessed eligibility, and randomized the participant. Afterwards, we activated a registration account through which we collected all subsequent data remotely via sensors. Participants assigned to the non-control arms were then introduced to the intelligent agents developed for their arm—“MyCoach” for the voice assist arm, and “SmartText” for the text messaging arm. Information about the participants, baseline date, and anticipated date to start the intervention were recorded. During the same visit, a wearable sensor (Fitbit Charge 2 HR) was provided to participants in all three arms with instructions on installation, charging, and use. Each wearable sensor had a serial number linking it to a research Application Programming Interface (API) that enabled data monitoring and transfer minute by minute.

Data were collected from the server, automatically recorded, and stored in a secure database; hence, it was unnecessary for the participant to return in-person the wearable device to download data. The wearable sensor transmitted 5 weeks’ worth of physical activity data of each participant—1-week baseline physical activity data, and 4 weeks of intervention physical activity data. The wearable sensor also served as an extension for the coaching agents to learn about the participant’s physical activity behavior.

### Randomization

We utilized Stratified Permuted Block Randomization to account for factors that may influence the study outcome such as age, sex, and body mass index. There were three allocations A, B, or C. We used 6 blocks of size 3. Blocks were concealed in smart form (Microsoft 365 Enterprise/Excel 2109) to eliminate guessing. We developed an automated tool to general stratum and blocks using Microsoft smart form, which effectively concealed the sequence within each block. At the randomization visit, each participant reviewed and signed the IRB-approved written consent form, then the Study Coordinator conducted the randomization assignment using www.random.org.

### Interventions

The trial tested two interventions compared to a control condition: (1) on-demand, AI coaching using interactive digital voice assist via Amazon Echo smart speaker, termed “MyCoach,” and (2) autonomous, data-driven smart text messaging via mobile phone, termed “SmartText. The third arm, “control”, received printed written information, specifically, a NCI publication about the benefits of physical activity for cancer survivors that recommended 10,000 steps per day of physical activity. In addition, the same publication was sent electronically via email after the end of baseline period to the control arm.

#### Voice-assisted AI “MyCoach” intervention

Participants in this intervention received personalized physical activity coaching via digital voice technology between the agent (MyCoach, delivered through the Amazon Echo/Alexa smart speaker) and the participant^18^. On the last day of the baseline period, a study team member visited the participant, installed an Amazon Echo device in the home, and gave instructions on how to use the voice technology. The participant had to interact with MyCoach®, via the smart speaker, to seek coaching; therefore, the intervention intensity and frequency was dependent on the participant’s intention and motivation to seek coaching. MyCoach used reinforced recommendation system to learn about the participant behavior and general responses to maximize rewards, some based on achieving the physical activity goal at least 10,000 steps per day. To enable reward feedback, the wearable sensor provided real-time data to MyCoach. The connection between the wearable sensor (Fitbit Charge HR2) and MyCoach was established using voice technology during installation of the smart speaker. MyCoach and its related databases were hosted on a secure local server at Johns Hopkins University. MyCoach design and how the intervention worked is displayed in Supplementary Fig. 1. Further details are published^16^.

#### Autonomous smart testing “SmartText” intervention

Participants in this arm received personalized physical activity coaching via text messages. After baseline, the participants received three messages each day. The text messages were initiated by an autonomous agent^19^, termed SmartText, a goal-based agent designed to progressively increase physical activity. The agent selects and then modifies the message content after considering an individual participant’s schedule, anthropometric measures, wearable sensor feedback, personal preferences, and progress over time. All computations were performed on a secure server, which received minute-by-minute data from the wearable sensors and combined it with the other participant data to formulate messages. The agent (SmartText) acted as a unidirectional coach, i.e., the agent sent messages to the participant, but the participant could not communicate with the agent. SmartText design and how the intervention worked is displayed in Supplementary Fig. 2. Further details are published^16^.

MyCoach and SmartText intervention uses a recommendation system (AI-agent) to provide coaching, but the interventions differed substantively^16^. SmartText used supervised goal-based model, while MyCoach uses an unsupervised goal base model with a reward condition(s). The time-specific parameter in MyCoach is influenced by priors, the user’s intent, and the physical activities captured by the wearable sensor. The model is connected to the Alexa voice interface using a proprietary Amazon platform (Alexa console). Response selection (messaging) is done by the agent using resource data bank. The SmartText arm message formulations were unidirectional, while the messaging formulation for MyCoach is bidirectional since it is dictated by the participant’s intent. The wearable sensor served as an extension for the agent/model to get feedback.

### Blinding

Due to the nature of the study interventions, study participants were not blinded. However, outcome ascertainment was blinded, given that the sensor collected and transferred data automatically.

### Study outcome

The aim of this study is to assess the effectiveness of different technological approaches in increasing physical activity among overweight and/or obese cancer survivors captured by wearable sensors. The primary outcome is the percentage of change in daily steps from the 1-week baseline to the end of the 4-week intervention period. Participant wear-time is validated using the heart rate sensor readings in the wearable device. For more details, please check the published protocol^16^.

### Data analysis and sample size

The primary endpoint was change in the average number of daily steps from baseline to the end of follow-up. An intention-to-treat analysis was used to determine the effects of each intervention compared to control and to compare the two active interventions (MyCoach and SmartText). Mean steps per day for baseline was calculated from 7 days of baseline data. Mean steps per day during follow-up was calculated from 4 weeks of intervention data. We computed average change in steps before and after intervention within each arm using a regression model in which *B*_0_ represents the step count per day, and *B*_1_ represents the regression coefficient of the two-sided *t* test by the stage of the study (baseline/follow-up) among the participants in each arm. A cluster by unique participant ID term was included in the regression model to treat each individual participant independently. The change in changes across arms was then computed by fitting a multiple linear regression model in which *B*_0_ represents the step count per day for each participant, *B*_1_ represents the trial period, and *B*_2_ represents the interaction term by period/arm. Change from baseline to follow-up in the control arm was the reference in the regression models. We adjusted for baseline and clustered by participant ID. Finally, the same outcomes were assessed using data from the final week of follow-up only. All baseline and follow-up summary measures are reported as mean and SD, and summary outcomes as mean with 95% CIs and *P* values.

Of the 1470 total expected person-days in the trial, 34 person-days (2.3%) were missing across the 3 study arms. Some were due to occasional synchronization issues of the wearable devices; for religious purposes, one participant specified days on which the wearable device would not be activated. Missing person-days were imputed with the average steps from the valid person-days. As for process measures, we reported measures of adherence by study arm: (1) number of participants in control arm that opened the written publication provided by email; (2) number of daily messages sent and received by each participant in SmartText arm; and (3) average number of daily interaction sessions in the MyCoach arm.

The sample size of 39 participants in total (13 per arm) was sufficient to detect a 2000 average steps/day, between-arm difference, assuming a standard deviation of 1800 steps/day, with power of 0.80 and a 2-sided alpha of 0.05^20,21^. To account for participant dropout, 42 participants were recruited. We used SQL to query our server, Python 3.7 to prepare the master data frame, and Stata/SE 15.1 for the statistical analysis.

### Reporting summary

Further information on research design is available in the Nature Research Reporting Summary linked to this article.

## Supplementary information

Supplementary Information

Reporting Summary

## Data Availability

The deidentified data are available from the corresponding author upon reasonable request.

## Code availability

Access to data collection codes can be obtained from the product websites: Fitbit API: https://dev.fitbit.com/build/reference/web-api/. Amazon Alexa APIs: https://developer.amazon.com/en-US/alexa/alexa-skills-kit/get-deeper/dev-tools-skill-management-api.
