# Supplementary material for: Randomized trial of two artificial intelligence coaching interventions to increase physical activity in cancer survivors
Source: NPJ Digit Med. 2021 Dec 9;4:168. doi: 10.1038/s41746-021-00539-9 (PMC8660785; doi:10.1038/s41746-021-00539-9)
Supplement: Supplementary file 1 — Supplementary Information [file 41746_2021_539_MOESM1_ESM.pdf]

Supplemental materials

Supplemental Figure 1: MyCoach design

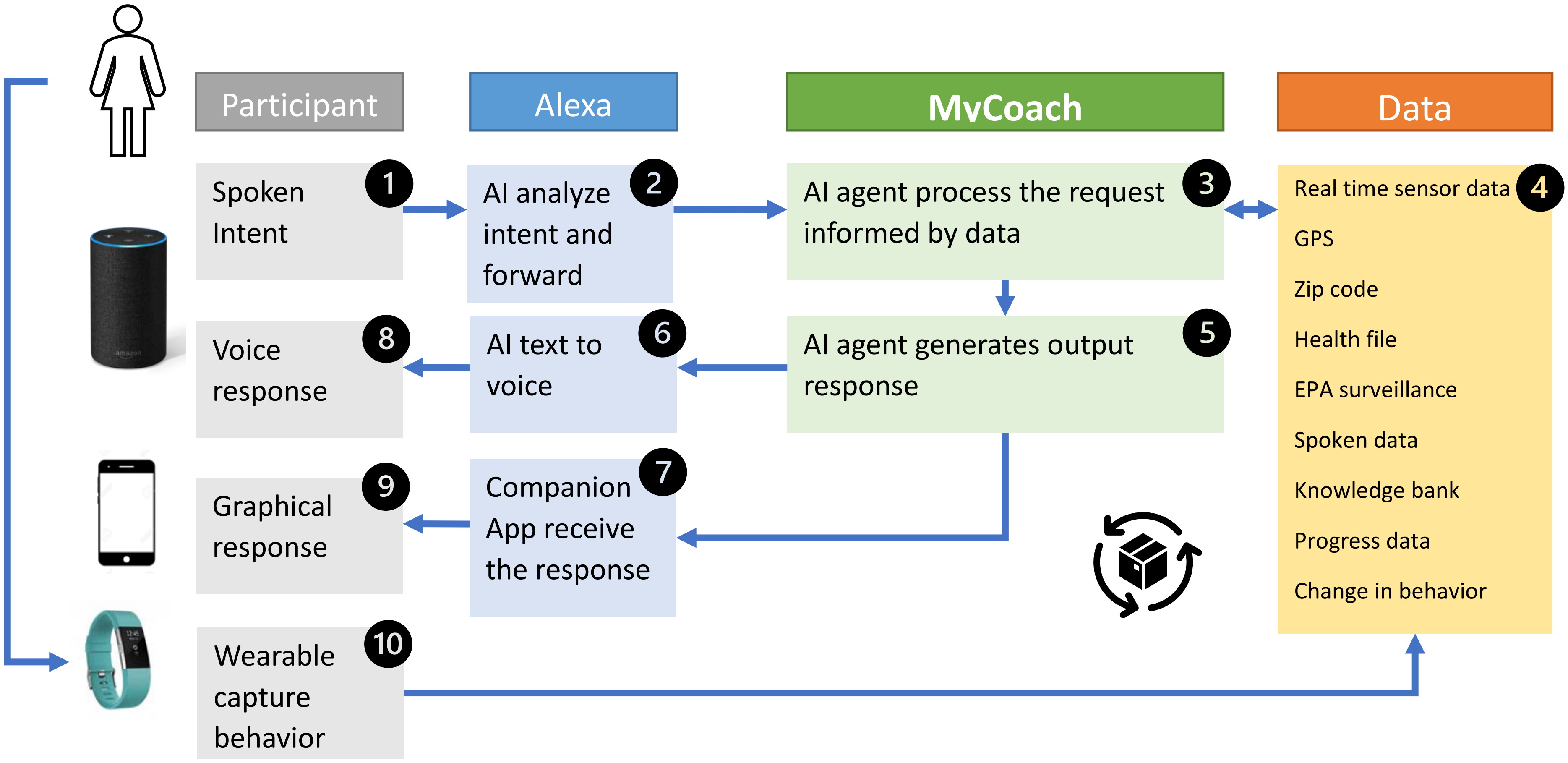

Supplemental Figure 2: SmarText design

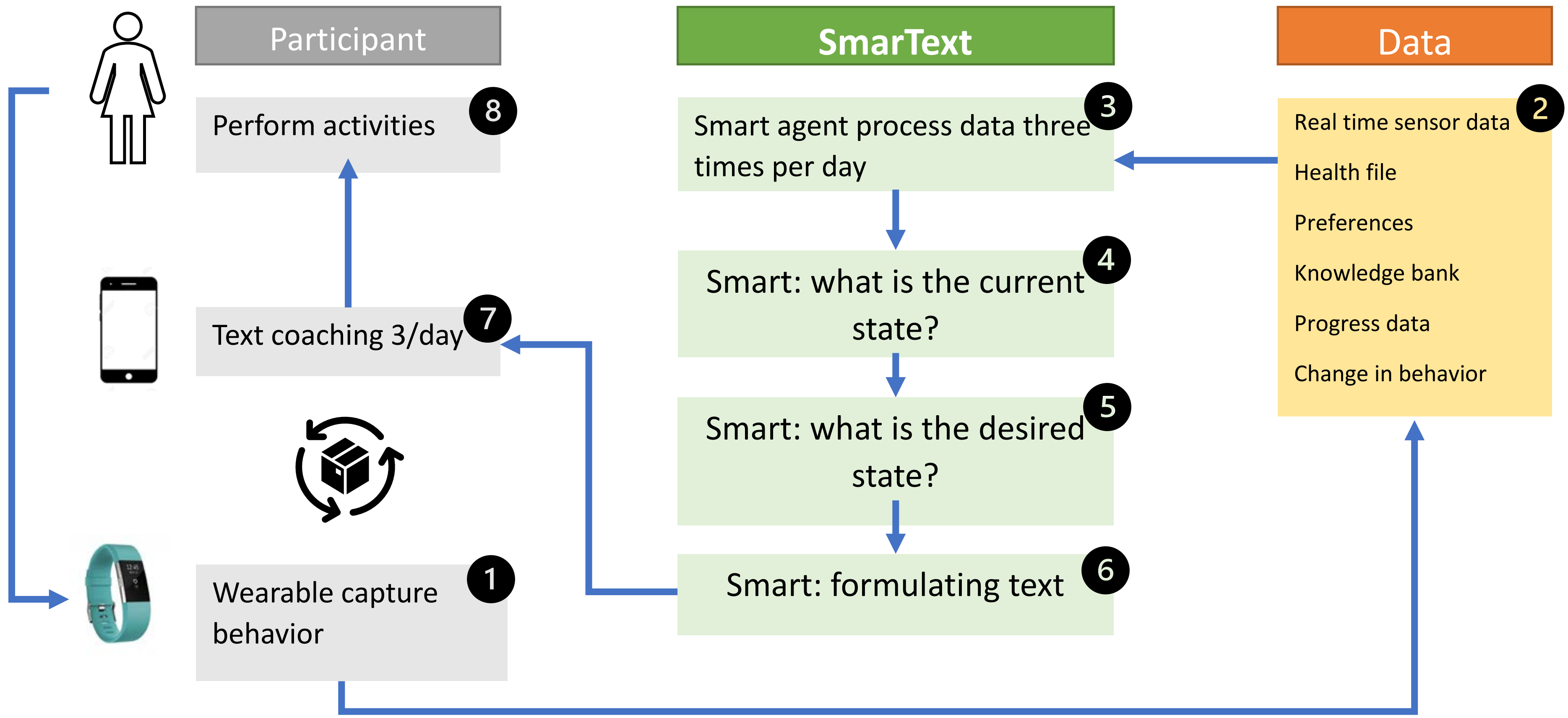

Supplemental Figure 3: CONSORT Flow Diagram for PATH Trial

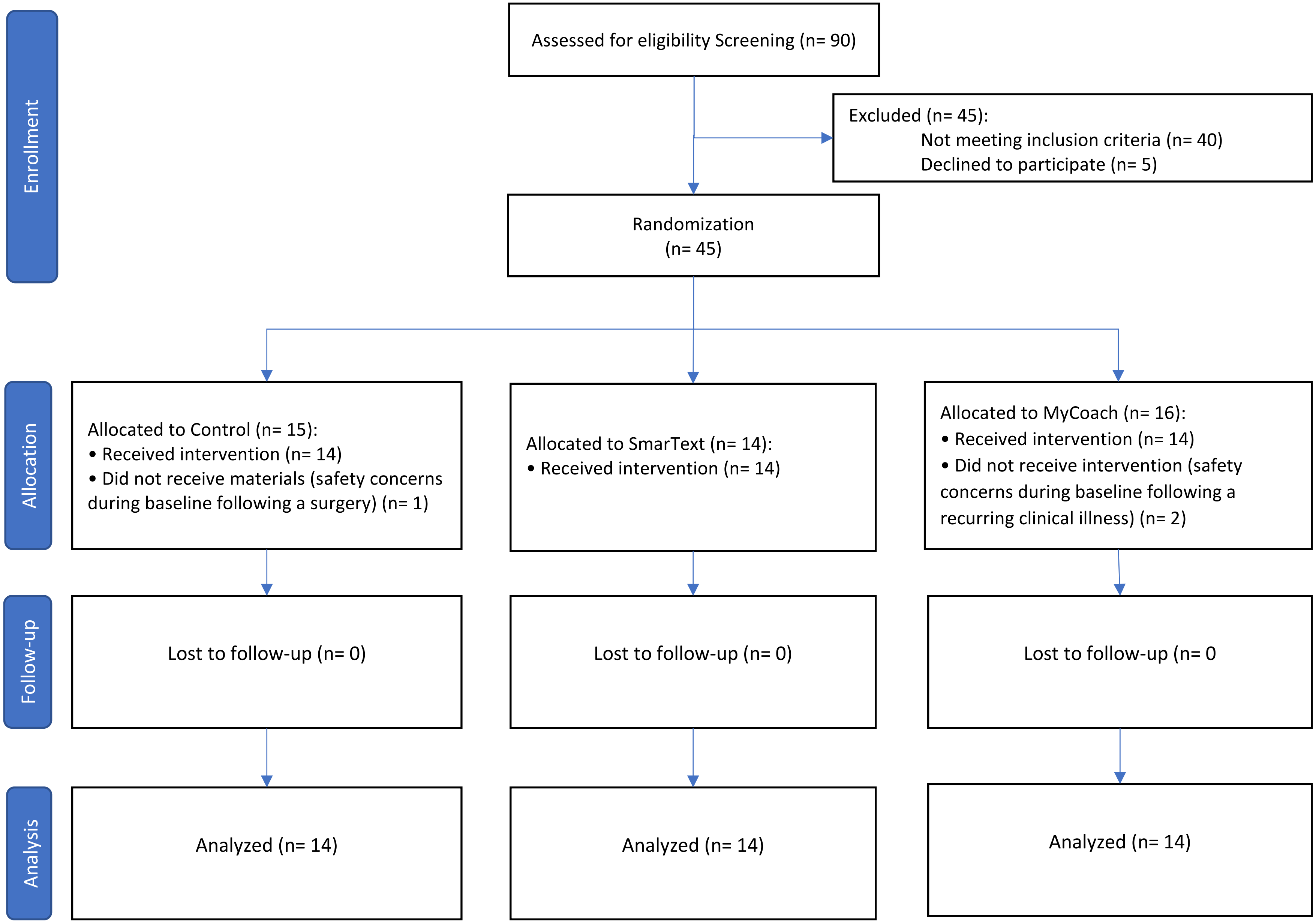

Supplemental Table 1: Summary comparison of each arm in the trial

#		Control	SmartText	MyCoach
1	Approach	Print and electronic educational materials about the benefit of physical activities for cancer survivors	Autonomous text messages	On demand coaching via voice assist
2	Contents	NCI educational materials	Behavioral coaching contents developed by the study team	Behavioral coaching contents developed by the study team
3	Coaching messages/responses	NCI recommendation on physical activities	Autonomous formulation by the goal-based agent	On demand formulation by unsupervised goal-based agent
4	Frequency	One time	Three times per day	On demand based on user's intent
5	Data collection	Sensor	Sensor	Sensor
6	Recommended target for physical activities	10,000 steps per day	10,000 steps per day	10,000 steps per day

Supplemental List: Additional data collected during the trial

- total number and duration of activity bouts (defined as 3 minutes or more of uninterrupted activity); 2)
- fragmentation indices (counting transitions between active/inactive periods) and variability indices (counting the magnitude of transitions between different levels of physical activity)
- daily diurnal patterns of activity that model patterns in minute-by-minute profiles
- heart rate and sleep data were captures. Subjected to additional user's permission to share.
