# Supplementary material for: Randomized trial of two artificial intelligence coaching interventions to increase physical activity in cancer survivors
Source: NPJ Digit Med. 2021 Dec 9;4:168. doi: 10.1038/s41746-021-00539-9 (PMC8660785; doi:10.1038/s41746-021-00539-9)
Supplement: Supplementary file 2 — Reporting Summary [file 41746_2021_539_MOESM2_ESM.pdf]

nature portfolio

Corresponding author(s): Ahmed Hassoon

Last updated by author(s): 10/11/2021

Reporting Summary

Nature Portfolio wishes to improve the reproducibility of the work that we publish. This form provides structure for consistency and transparency in reporting. For further information on Nature Portfolio policies, see our Editorial Policies and the Editorial Policy Checklist.

Statistics

For all statistical analyses, confirm that the following items are present in the figure legend, table legend, main text, or Methods section.

n/a	Confirmed	
☐	☒	The exact sample size (n) for each experimental group/condition, given as a discrete number and unit of measurement
☒	☐	A statement on whether measurements were taken from distinct samples or whether the same sample was measured repeatedly
☐	☒	The statistical test(s) used AND whether they are one- or two-sided<br>*Only common tests should be described solely by name; describe more complex techniques in the Methods section.*
☐	☒	A description of all covariates tested
☐	☒	A description of any assumptions or corrections, such as tests of normality and adjustment for multiple comparisons
☐	☒	A full description of the statistical parameters including central tendency (e.g. means) or other basic estimates (e.g. regression coefficient) AND variation (e.g. standard deviation) or associated estimates of uncertainty (e.g. confidence intervals)
☒	☐	For null hypothesis testing, the test statistic (e.g. F, t, r) with confidence intervals, effect sizes, degrees of freedom and P value noted<br>*Give P values as exact values whenever suitable.*
☒	☐	For Bayesian analysis, information on the choice of priors and Markov chain Monte Carlo settings
☒	☐	For hierarchical and complex designs, identification of the appropriate level for tests and full reporting of outcomes
☒	☐	Estimates of effect sizes (e.g. Cohen's d, Pearson's r), indicating how they were calculated

Our web collection on statistics for biologists contains articles on many of the points above.

Software and code

Policy information about availability of computer code

Data collection	Fitbit API: https://dev.fitbit.com/build/reference/web-api/<br>Amazon Alexa APIs: https://developer.amazon.com/en-US/alexa/alexa-skills-kit/get-deeper/dev-tools-skill-management-api
Data analysis	Stata 15, Python 3.7 Panda package

For manuscripts utilizing custom algorithms or software that are central to the research but not yet described in published literature, software must be made available to editors and reviewers. We strongly encourage code deposition in a community repository (e.g. GitHub). See the Nature Portfolio guidelines for submitting code & software for further information.

Data

Policy information about availability of data

All manuscripts must include a data availability statement. This statement should provide the following information, where applicable:

- Accession codes, unique identifiers, or web links for publicly available datasets
- A description of any restrictions on data availability
- For clinical datasets or third party data, please ensure that the statement adheres to our policy

The deidentified data are available from the corresponding author upon reasonable request and JHU IRB approval

Field-specific reporting

Please select the one below that is the best fit for your research. If you are not sure, read the appropriate sections before making your selection.

☒ Life sciences ☐ Behavioural & social sciences ☐ Ecological, evolutionary & environmental sciences

For a reference copy of the document with all sections, see nature.com/documents/nr-reporting-summary-flat.pdf

Life sciences study design

All studies must disclose on these points even when the disclosure is negative.

Sample size	42
Data exclusions	N/A
Replication	N/A
Randomization	We utilized Stratified Permuted Block Randomization to account for factors that may influence the study outcome such as age, sex, and body mass index. We developed an automated tool to general stratum, and blocks using Microsoft smart form, which effectively concealed the sequence within each block. At the randomization visit, each participant reviewed and signed the IRB approved written consent form, then the Study Coordinator conduct the randomization assignment using www.random.org
Blinding	Due to the nature of the study interventions, study participants were not blinded. However, outcome ascertainment was blinded, given that the sensor collected and transferred data automatically

Reporting for specific materials, systems and methods

We require information from authors about some types of materials, experimental systems and methods used in many studies. Here, indicate whether each material, system or method listed is relevant to your study. If you are not sure if a list item applies to your research, read the appropriate section before selecting a response.

Materials & experimental systems

n/a	Involved in the study	
☒	☐	Antibodies
☒	☐	Eukaryotic cell lines
☒	☐	Palaeontology and archaeology
☒	☐	Animals and other organisms
☐	☒	Human research participants
☒	☐	Clinical data
☒	☐	Dual use research of concern

Methods

n/a	Involved in the study	
☒	☐	ChIP-seq
☒	☐	Flow cytometry
☒	☐	MRI-based neuroimaging

Human research participants

Policy information about studies involving human research participants

Population characteristics	We recruited overweight or obese cancer survivors. Mean (SD) age was 62.1(9.8) years and mean (SD) BMI was 32.9(5.0). The majority of participants (85.7%) had stage 1 or 2 breast cancer. Baseline characteristics were similar in the study arms.
Recruitment	Participants are recruited using both passive and active strategies. In the passive strategy, the study team distributes flyers at the Johns Hopkins outpatient oncology clinics, patient education rooms, survivorship clinics, and survivorship meetings to spread study awareness among clinic staff, particularly nurse educators and managers. With the active strategy for recruitment, the Epic reporting function is used to generate patient lists of those who match the screening criteria at selected Johns Hopkins clinics in Maryland. Screening for existing cancer patients takes place at the outpatient clinics, by specific providers and on follow-up weekly appointments only; potential participants must have a prior diagnosis of a cancer of interest, a BMI of 25 or above, and reside in the state of Maryland.
Ethics oversight	Johns Hopkins Medicine IRB

Note that full information on the approval of the study protocol must also be provided in the manuscript.
